# Effectiveness of the Essential Coaching Postpartum Digital Health Solution on Parenting Self-Efficacy, Mental Health, Well-Being, and Parenting Outcomes: Protocol for a Randomized Controlled Trial

**DOI:** 10.2196/78209

**Published:** 2025-10-17

**Authors:** Justine Dol, Christine T Chambers, Jennifer A Parker, Melissa Brooks, Cindy-Lee Dennis, Daniel Seguin, Jennifer M Goldberg, Brad Hughes, Teresa Reese, Greg Richard, Kate Calnan

**Affiliations:** 1 IWK Health Halifax, NS Canada; 2 Department of Psychology and Neuroscience Faculty of Science Dalhousie University Halifax, NS Canada; 3 Department of Pediatrics Faculty of Medicine Dalhousie University Halifax, NS Canada; 4 Department of Obstetrics and Gynecology Faculty of Medicine Dalhousie University Halifax, NS Canada; 5 Lawrence S Bloomberg Faculty of Nursing University of Toronto Toronto Canada; 6 Lunenfeld-Tannenbaum Research Institute Mount Sinai Hospital Toronto, ON Canada; 7 Department of Psychology Mount Saint Vincent University Halifax, NS Canada; 8 McMaster Midwifery Research Centre McMaster University Hamilton, ON Canada; 9 Patient Partner Halifax, NS Canada

**Keywords:** mobile health, mHealth, parents, SMS text message, postpartum

## Abstract

**Background:**

The transition to parenthood requires adjustments to new norms and expectations. While there is significant focus on the parenting transition for birthing parents (persons with a uterus), nonbirthing parents (parents who do not give birth, including fathers, gender-diverse parents, adoptive parents, or nongestational coparents) are less supported. Preventive education via SMS text messaging can fill a health service gap and provide timely, standardized, evidence-based information. The Essential Coaching Postpartum program includes 6 weeks of evidence-based daily SMS text messages on topics such as infant care, normal development, parental mental health, and self-care, with streams designed for birthing parents (Essential Coaching for Every Mother) and nonbirthing parents (Essential Coaching for Every Partner).

**Objective:**

The primary objective of this study is to compare the effectiveness of Essential Coaching Postpartum to standard care on parenting self-efficacy, mental health (ie, depression, anxiety, and stress), well-being (ie, general distress and sleep), and parenting outcomes (ie, relationship satisfaction and coparenting) among first-time nonbirthing parents in Nova Scotia, Canada. The secondary objective is to compare the effectiveness of Essential Coaching Postpartum to standard care among first-time birthing parents in Nova Scotia. The exploratory objective is to compare the effectiveness of Essential Coaching Postpartum to standard care among first-time parent dyads (ie, both birthing parent and nonbirthing parent) in Nova Scotia.

**Methods:**

This will be a randomized controlled trial. A total of 166 first-time birthing parents and 166 first-time nonbirthing parents from Nova Scotia will be recruited and randomly assigned to the intervention or control arm. The intervention arm will receive standard care plus the Essential Coaching Postpartum digital health solution, which consists of twice-daily SMS text messages in the first 3 weeks and daily messages in the next 3 weeks. The control group will receive standard care. Messages are personalized based on the infant’s age and the parent’s self-selected feeding preference (ie, breast or chest feeding, formula feeding, or combination feeding). The first message is sent on the second evening after birth, with parents being eligible to enroll up to 7 days post partum. Participants will complete questionnaires assessing parenting self-efficacy, mental health, well-being, and parenting outcomes at baseline (enrollment after birth), 6 weeks post partum (after the intervention), and 6 months post partum (follow-up).

**Results:**

Recruitment for this study started in June 2025 and is currently ongoing. As of September 15, 2025, 11 birthing and 5 nonbirthing parents have been recruited.

**Conclusions:**

We anticipate that providing parents with SMS text messages (ie, Essential Coaching Postpartum) during the 6-week postpartum period to complement standard care will improve parenting self-efficacy, mental health, well-being, and parenting outcomes.

**Trial Registration:**

ClincalTrials.gov NCT06996067; https://www.clinicaltrials.gov/study/NCT06996067

**International Registered Report Identifier (IRRID):**

PRR1-10.2196/78209

## Introduction

### Background

The transition to parenthood is a transformative period that requires physical, emotional, and social adjustments to new norms and expectations. While there is significant focus on the transition for birthing parents (persons with a uterus) [1], nonbirthing parents (parents who do not give birth, including fathers, sexual-minority parents, adoptive parents, or nongestational coparents) are less supported in the transition to parenthood [2,3]. Perinatal mental health and psychosocial adjustment challenges are common among both parents, which can have significant impacts on parenting relationships and child outcomes [4,5]. Higher parenting self-efficacy, defined as a parent’s perception of their ability to execute tasks associated with raising a child [6], has been associated with improvement in many outcomes for both parents and children, including lower parental anxiety and depression and fewer behavioral problems in children [7]. Even in uncomplicated pregnancies and births, parents may struggle with developing their parenting self-efficacy [8] and report anxiety and depression symptoms [9-12]. Poor parental mental health is known to be comorbid and can adversely impact the interparental relationship, leading to relationship dysfunction and negatively impacting child outcomes [13]. By contrast, high parental self-efficacy in one parent is associated with higher parenting self-efficacy in the other [14]. Thus, it is essential to identify ways to support all parents during the early postpartum period to improve individual, couple, and family outcomes.

In Canada, recommendations for postnatal care vary by province, with no standardization of care, education, or support across the country [15]. In Nova Scotia, the recommendations for birthing parents are to have a comprehensive postpartum assessment with their health care provider at approximately 6 to 8 weeks after birth [15]. While there is focus on family-centered care, there are no specific follow-up recommendations for the nonbirthing parent, leading to a significant gap in support. Families should be offered postpartum depression screening at 2 weeks post partum, 6 to 8 weeks post partum, and 4 months post partum [15]. However, this does not always occur in practice [16].

The lack of formal support during the postpartum period is a sharp shift from the intense monitoring that occurs near the end of pregnancy, when birthing people often meet with health care providers on a weekly basis [17]. After a person gives birth, this access to perinatal follow-up care is reduced significantly to only once or twice in the first year, with the primary assessment occurring typically at approximately 6 weeks post partum [18]. The amount and type of care a family receives post partum may depend on whether their obstetric care provider was a physician or a midwife, whether they have a primary care provider, whether they are considered to have high needs, or whether they have identified health concerns for themselves or their infant [15]. The care for a nonbirthing parent during this period is often nonexistent, with nonbirthing parents reporting feeling invisible to health care providers [19], leading to a gap in access to information and support during this critical period for new parents.

An innovative strategy that can be used to support parents is mobile health, defined as the use of mobile devices to transmit health content and services [20]. Parents already use their mobile phones to access internet-based information during the perinatal period [21,22]. SMS text messaging can be an ideal strategy for reaching and supporting parents because it allows access to information anytime, anywhere; moreover, the messages can deliver evidence-based information consistent with the infant’s developmental age [23]. Preventive education via SMS text messaging can fill a health service gap and provide timely, standardized, evidence-based information.

To address this need, our team developed and evaluated an SMS text messaging program, Essential Coaching for Every Mother, in which we demonstrated improved parenting self-efficacy and reduced postpartum anxiety in first-time birthing parents [24-26]. A limitation of the original program was that it focused only on birthing parents, excluding nonbirthing parents. Thus, we recently developed Essential Coaching for Every Partner to focus on nonbirthing parents, which complements the information provided in Essential Coaching for Every Mother by delivering evidence-based information directly to nonbirthing parents via SMS text messaging during the first 6 weeks of the postpartum period [27]. The Essential Coaching Postpartum program (inclusive of all types of parents) includes 6 weeks of evidence-based daily SMS text messages on topics such as infant care, normal development, parental mental health, and self-care and was designed in collaboration with parents and postpartum health care providers based on evidence-based information [28-31]. The goal of Essential Coaching Postpartum is not to replace face-to-face contact with health care providers but rather be an adjunct to standard care with specialized information targeting the unique needs of new parents. The aim of this study is to evaluate the effectiveness of Essential Coaching Postpartum on nonbirthing parents, birthing parents, and parent dyads in Nova Scotia.

### Objectives

The primary objective is to compare the effectiveness of Essential Coaching Postpartum with standard care on parenting self-efficacy, mental health (ie, depression, anxiety, and stress), well-being (ie, general distress and sleep), and parenting outcomes (ie, relationship satisfaction and coparenting) among first-time nonbirthing parents in Nova Scotia.

The secondary objective is to compare the effectiveness of Essential Coaching Postpartum with standard care on parenting self-efficacy, mental health, well-being, and parenting outcomes among first-time birthing parents in Nova Scotia.

The explorative objective is to compare the effectiveness of Essential Coaching Postpartum with standard care on parenting self-efficacy, mental health, well-being, and parenting outcomes among first-time parent dyads (ie, both birthing parent and nonbirthing parent) in Nova Scotia.

### Hypotheses

#### Primary Hypothesis

Our primary hypothesis is as follows: nonbirthing parents who receive Essential Coaching Postpartum will have higher parenting self-efficacy, better mental health (ie, lower scores for depression, anxiety, and stress), greater well-being (ie, lower distress scores and better sleep scores), and higher parenting outcomes (ie, higher relational satisfaction and coparenting) at 6 weeks post partum than those who receive standard care.

#### Secondary Hypotheses

Our secondary hypotheses are as follows: (1) birthing parents who receive Essential Coaching Postpartum will have higher parenting self-efficacy, better mental health, greater well-being, and greater parenting outcomes at 6 weeks post partum than those who receive standard care; (2) nonbirthing parents who receive Essential Coaching Postpartum will have higher parenting self-efficacy, better mental health, greater well-being, and greater parenting outcomes at 6 months post partum than those who receive standard care; and (3) birthing parents who receive Essential Coaching Postpartum will have higher parenting self-efficacy, better mental health, greater well-being, and greater parenting outcomes at 6 months post partum than those who receive standard care.

#### Exploratory Hypotheses

Our exploratory hypotheses are as follows: (1) parent dyads who receive Essential Coaching Postpartum will have higher parenting self-efficacy, better mental health, greater well-being, and greater parenting outcomes at 6 weeks post partum than those who receive standard care; and (2) parent dyads who receive Essential Coaching Postpartum will have higher parenting self-efficacy, better mental health, greater well-being, and greater parenting outcomes at 6 months post partum than those who receive standard care.

## Methods

### Study Design

A 2-group, parallel-arm randomized controlled trial (RCT) will be conducted to evaluate the effectiveness of Essential Coaching Postpartum on parenting self-efficacy, mental health (ie, depression, anxiety, and stress), well-being (ie, general distress and sleep), and parenting outcomes (ie, relationship satisfaction and coparenting) among first-time birthing and nonbirthing parents across Nova Scotia. The intervention will be compared to standard care that is currently being provided across Nova Scotia. This study follows a protocol similar to the original evaluation of Essential Coaching for Every Mother [32].

### Ethical Considerations

This study has been approved by the IWK Health Research Ethics Board (1031429) and is registered with ClinicalTrials.gov (NCT06996067). The CONSORT-EHEALTH (Consolidated Standards of Reporting Trials of Electronic and Mobile Health Applications and Online Telehealth) checklist [26] will be followed to enhance reporting quality for this eHealth intervention trial.

All participants will be asked to provide consent via REDCap (Research Electronic Data Capture; Vanderbilt University) [33], a secure web application for building and managing web-based surveys and databases, followed immediately by the baseline survey. Participants who do not complete the consent form or baseline survey will be considered to have withdrawn from the study.

To maintain confidentiality, all study data will be identified using a study number unique to each participant. Contact information will be collected via TextIt (Nyaruka) [34], a platform used to create and manage 2-way mobile messaging applications, which will be kept separate from survey data collected via REDCap to protect confidentiality.

To encourage survey completion, participants will be offered an honorarium for their time. Electronic gift cards worth CAD $5 (US $3.62) will be provided for each completed survey at baseline, 6 weeks post partum, and 6 months post partum. Participants who complete all 3 surveys will be entered into a draw for an electronic gift card worth CAD $250 (US $181.10). Four separate draws will be held: one each for the birthing parents in the control and intervention groups and one each for the nonbirthing parents in the control and intervention groups.

### Setting

This study will be conducted in Nova Scotia. In 2023, there were 6930 births in the province, with 3464 occurring in Halifax (primarily at IWK Health) [35]. There is a lack of standardized, universal postpartum support for new parents across the province. The Public Health Early Years program provides prenatal and postnatal support to all new parents giving birth in Nova Scotia. The *Loving Care* book series, developed by the Nova Scotia Department of Health and Wellness in partnership with Public Health Services, is offered after birth to provide information to new families about what to expect [28,29]. Families may also be offered a postnatal home visit with a nurse to provide support, including community and health referrals, guidance on infant care, and bonding. If more support is indicated, families are offered the enhanced home visiting program, which offers home visits from a community home visitor until the child turns 3 years of age [36]. Furthermore, 16% of people in the province do not have a primary care provider, resulting in a significant and growing number of newborns being referred to mobile “unattached newborn clinics” to receive care and support during the postpartum period [37,38].

### Patient Partnership

Four patient partners from diverse backgrounds and experiences as birthing and nonbirthing parents are involved in all aspects of planning, executing, and disseminating the work, following best practices in patient partnership.

### Eligibility Criteria

Participants will be recruited prenatally and postnatally. To pre-enroll prenatally, eligible birthing parents will (1) be pregnant (>28 wk) with their first child, (2) understand English, (3) have a mobile phone with SMS text messaging functionality and a data plan, and (4) live in Nova Scotia. Eligible nonbirthing parents will (1) have a pregnant (>28 wk) partner (ie, the birthing parent) expecting their first child or will be expecting their first child via an alternative method (eg, adoption or surrogacy), (2) understand English, (3) have a mobile phone with SMS text messaging functionality and a data plan, and (4) live in Nova Scotia. Participants who pre-enroll prenatally will only be considered enrolled and assigned a study number once they text “birth” to the study mobile phone number and are randomly assigned.

To enroll postnatally, eligible birthing parents will (1) be early postpartum (<7 d) with their first child, (2) understand English, (3) have a mobile phone with SMS text messaging functionality and a data plan, and (4) live in Nova Scotia. Eligible nonbirthing parents will (1) be early postpartum (<7 d) with their first child, (2) understand English, (3) have a mobile phone with SMS text messaging functionality and a data plan, and (4) live in Nova Scotia.

Participants (both birthing and nonbirthing parents) will be excluded if they (1) have a newborn who dies or is expected to die before leaving the hospital; (2) have no access to a mobile phone, either personal or shared; (3) are unwilling to receive SMS text messages; (4) decline participation or withdraw; or (5) participated in a previous iteration of this project (eg, development or evaluation). Parents are not required to participate as dyads to be inclusive of all family compositions; however, involvement of the other parent, if applicable, will be encouraged. Participants who do not fully complete the baseline survey will be considered withdrawn from the study. If the participant was randomly assigned to the intervention, they will continue to receive the messages, but no further data will be collected from them.

### Recruitment

Parents will be recruited through social media, posters, and in-person outreach (eg, clinics and the family newborn unit). Social media advertisements will be used to recruit parents who are more than 28 weeks pregnant or within 7 days post partum; in addition, parents will be recruited on the birth unit and the family newborn unit at IWK Health, where print posters will be displayed in the patient rooms. Additional outreach strategies may include promotion through public health services, family resource centers, media campaigns, other hospitals across Nova Scotia, and word of mouth. A research team member may also approach families at relevant clinics or units at IWK Health if a nurse within their circle of care has received approval from the family to be approached for research. Participants who contact us by texting “pregnant” to the study mobile phone number when they are less than 28 weeks pregnant will be sent a follow-up message once they reach 28 weeks, until the required sample size is achieved.

### Screening and Baseline

Interested participants will complete a standardized SMS text message script for the eligibility screening process. During prenatal screening, participants will be assessed for preliminary eligibility, and those who qualify will be asked to text the study mobile phone number within 7 days of giving birth to be enrolled. At this stage, participants will be encouraged to provide their partner’s mobile phone number, if applicable, so that the partner’s interest can be assessed. Once a birthing person gives birth or enrolls post partum, they will complete additional screening questions to confirm eligibility.

After the birth of their child, all interested participants will complete the postpartum eligibility and randomization processes. During this stage, additional information will be collected, including the newborn’s name, date of birth, and feeding preference (ie, breast or chest feeding, formula feeding, or combination feeding). Participants will then be randomly assigned to the intervention or control group and asked to complete the consent form and baseline survey. If a parent dyad is participating, the nonbirthing partner will be sent an SMS text message after randomization to indicate their preferred type of feeding messages and to complete the consent form and baseline survey. Linked parent dyads will always be placed in the same intervention group. If dyad members complete the screening process separately and are inadvertently assigned to different groups, both parents will be moved to the intervention group.

### Randomization

Participants will be randomly assigned in a 1:1 ratio to either the intervention or control group using the TextIt platform’s “split randomly” function. This function distributes contacts equally across pathways, ensuring allocation to either the intervention or control condition. Randomization will occur only after a participant has given birth.

### Follow-Up Assessments

All participants will be asked to complete follow-up surveys at 6 weeks post partum and 6 months post partum. To encourage completion, reminder SMS text messages will be sent every other day for up to 2 weeks (a maximum of 6 reminders) or until the survey is submitted. After day 14, an email will be sent to participants who have not yet completed the survey as a final reminder. If no email address was provided, an extended SMS text message will be sent instead. Participants may also be contacted by phone to encourage survey completion within this 2-week window.

### Blinding

Due to the complex nature of Essential Coaching Postpartum, participants cannot be blinded and will be informed of their group allocation. To minimize blinding of personnel in relation to data collection, no hospital staff (eg, nurse midwives, physicians, and other health care providers) will be aware of participant involvement in the study. This is feasible because Essential Coaching Postpartum is delivered directly to participants’ personal mobile phones, with no in-person intervention. The research team will be aware of participant allocation and will track data collection but will not be involved in the randomization process, which occurs automatically through the TextIt platform. As all study procedures are conducted remotely through a preconfigured system, the research team has no direct interaction with participants, thereby reducing any risk of influencing participant responses.

### Sample Size

Sample size calculations were conducted with the assistance of a statistician for both birthing and nonbirthing parents to ensure adequate numbers for each group. As the parent dyad objective is exploratory, no formal calculation was performed for dyads. In the original RCT [25], the baseline parenting self-efficacy score for birthing parents was 35, which is considered clinically low. This value was used for this analysis. On the basis of the SD from the original RCT [25], an improvement of 4 points in the intervention group at 6 weeks is anticipated, which would no longer be classified as clinically low. A total sample size of 141 parents is therefore required, assuming 80% power and a 2-tailed α of .05. As no comparable evidence is available for nonbirthing parents, we will apply the same sample size estimation (n=141) for nonbirthing parents. Allowing for 15% attrition, we plan to recruit a minimum of 166 birthing parents and 166 nonbirthing parents.

### Program Content and Design

Essential Coaching Postpartum consists of 2 separate but related evidence-based programs: Essential Coaching for Every Mother, which provides SMS text messages for the birthing person, and Essential Coaching for Every Partner, which provides SMS text messages for the nonbirthing parent. Both were co-designed with parents and postpartum health care providers [24,27] and are aligned with the *Loving Care* book series [28-31]. Essential Coaching Postpartum includes 59 SMS text messages: 2 messages per day in the first 3 weeks and daily messages in the next 3 weeks. Topics include postpartum mental health, self-care, postnatal follow-up, feeding, infant concerns, cord care, well-baby care, normal development, crying, and safe sleep. Parents can choose to receive messages related to breast or chest feeding, formula feeding, or combination feeding and can change their preference at any time. Messages are timed according to the infant’s date of birth and personalized with the infant’s name. All messages are free to receive; however, optional embedded links may generate data charges if accessed.

All participants, regardless of group allocation, will receive a welcome message from Essential Coaching Postpartum as well as reminders to complete the surveys. Participants may withdraw from the study at any time by texting “stop” to the study mobile phone number, after which they will no longer receive messages or be asked to complete additional surveys.

### Intervention Group

**Figure 1:**
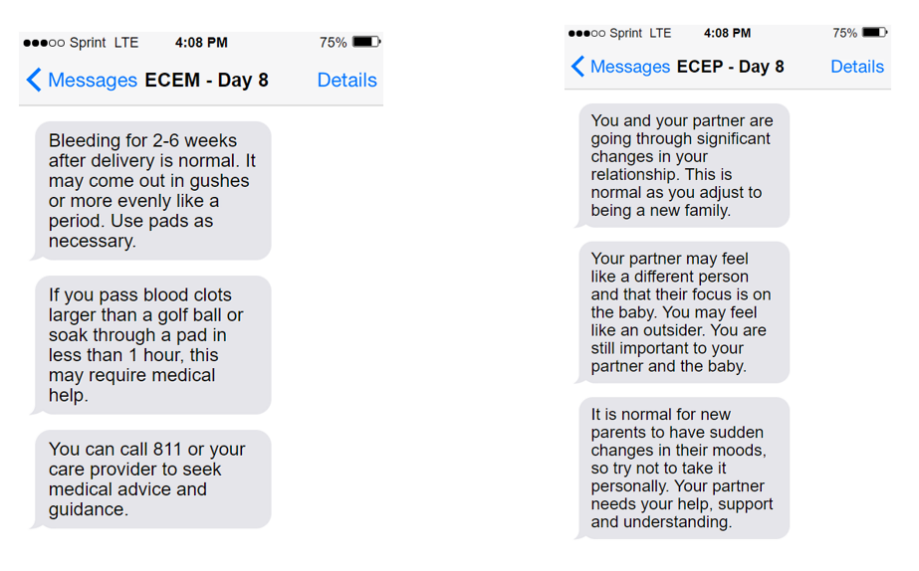
Essential Coaching for Every Mother (ECEM) and Essential Coaching for Every Partner (ECEP) example SMS text messages.

Participants will receive the standard education provided at the hospital where they give birth. After enrollment, participants in the intervention group will receive messages until 6 weeks post partum. Birthing parents will receive Essential Coaching for Every Mother, and nonbirthing parents will receive Essential Coaching for Every Partner. Figure 1 provides examples of messages from each program. Messages are automatically scheduled according to the infant’s date of birth, beginning on the second evening after birth. Participants who enroll later will receive messages starting from their enrollment date (eg, someone who enrolls on day 5 will not receive messages from days 2-4). TextIt will be used to program and schedule the messages, and Twilio (Twilio Inc) [39] will serve as the secure gateway for sending and receiving messages. Participants are not restricted from seeking additional postpartum support outside of the study. They will continue to have access to standard perinatal care, including mental health services available through primary care providers and community resources.

### Control Group

Participants will receive the standard education provided at the hospital in which they give birth. After enrollment, participants in the control group will not receive any Essential Coaching Postpartum SMS text messages. They are not restricted from seeking additional postpartum support outside of the study. They will continue to have access to standard perinatal care, including mental health services available through primary care providers and community resources. At enrollment, 6 weeks post partum, and 6 months post partum, participants will be sent an SMS text message with a link to complete the follow-up survey.

### Study Measures

Data will be collected from parents using self-reported web-based surveys administered through REDCap [33]. Table 1 outlines the timing of outcome collection. At baseline, demographics will be collected, including age, occupation, marital status, and educational level, as well as newborn information such as birth weight and delivery method. At 6 weeks post partum, a follow-up survey will be conducted to collect data on types of postpartum support sought and received, health concerns, and feeding behavior. All standardized questionnaires listed in Table 1 will be administered at baseline, 6 weeks post partum, and 6 months post partum.

**Table 1:** Timing of measurement and outcome measurement questionnaires.

Outcome domains and variables			Outcome measure		Baseline (enrollment after birth)		After the intervention (6 wk postpartum)		Follow-up (6 mo postpartum)
**Descriptive**									
	Demographics	Parent and infant characteristics^a^		✓					
**Effectiveness**									
	Parenting self-efficacy	Karitane Parenting Confidence Scale [40]		✓		✓		✓	
	Postpartum depression	Edinburgh Postnatal Depression Scale [41]		✓		✓		✓	
	Postpartum anxiety	Postpartum Specific Anxiety Scale [42]		✓		✓		✓	
	Postpartum stress	Postpartum Childcare Stress Checklist [43]		✓		✓		✓	
	General distress	Depression, Anxiety, and Stress Scale [44]		✓		✓		✓	
	Sleep	PROMIS^b^ Short Form Sleep-Related Impairment (4a) and PROMIS Short Form Sleep Disturbances (4a) [45]		✓		✓		✓	
	Coparenting	Brief Coparenting Relationship Scale [46]		✓		✓		✓	
	Relationship satisfaction	Couple Satisfaction Index-4 [47]		✓		✓		✓	
**Implementation**									
	Program satisfaction	Participant satisfaction survey and health service use^a^				✓			

^a^Researcher generated.

^b^PROMIS: Patient-Reported Outcomes Measurement Information System.

### Primary Outcomes

Parenting self-efficacy will be measured using the Karitane Parenting Confidence Scale [40]. This 15-item tool assesses perceived self-efficacy among parents of infants aged birth to 12 months of age and has acceptable internal consistency (Cronbach α=0.81) and test-retest reliability (*r*=0.88) [40]. A cutoff score of 39 or less (out of 45) indicates clinically low perceived parenting self-efficacy [40].

### Secondary Outcomes

Secondary outcomes will be collected using validated self-report measures, as follows:

Postpartum anxiety symptoms will be measured using the Postpartum Specific Anxiety Scale [42], a 51-item valid and reliable tool for assessing anxiety during the first 6 months post partum. A score of 112 or greater indicates clinical levels of anxiety [42].

Postpartum depression symptoms will be measured using the Edinburgh Postnatal Depression Scale [41]. A score greater than 9 (out of 30) suggests possible postpartum depression in both birthing and nonbirthing parents.

Postpartum stress will be measured using the Postpartum Childcare Stress Checklist [43], an 18-item scale assessing parental perceptions of childcare stress in the early postpartum period. Responses are summed to produce a score from 0 to 23, with higher scores indicating a higher level of postpartum childcare stress.

General distress will be measured using the Depression, Anxiety, and Stress Scale [44], a 21-item instrument that assesses the emotional states of depression, anxiety, and stress. Postnatal distress is classified by percentile scores, with total score (between 0 and 63) and a score for the three subscales (between 0 and 21), categorized into 5 severity ranges: normal, mild, moderate, severe and extremely severe, with higher scores indicated greater severity.

Sleep will be measured using the Patient-Reported Outcomes Measurement Information System (PROMIS) Short Form Sleep-Related Impairment (4a) and PROMIS Short Form Sleep Disturbances (4a) [45]. Sleep-related impairment measures perceptions of alertness, sleepiness, and tiredness during usual waking hours, as well as perceived functional impairments during wakefulness associated with sleep problems or impaired alertness. By contrast, sleep disturbance measures perceptions of sleep quality, sleep depth, and restoration associated with sleep. Higher scores indicate greater sleep-related impairment and disturbance.

Relationship satisfaction will be measured using the Couple Satisfaction Index [47], a 4-item scale assessing satisfaction in a relationship. Higher scores indicate greater satisfaction, whereas a score less than 13.5 is associated with notable dissatisfaction.

Coparenting will be measured using the Brief Coparenting Relationship Scale [46], a validated 15-item instrument assessing 5 coparenting dimensions of agreement: closeness, child exposure to conflict, coparenting undermining, endorsement of partner’s parenting, and labor division [46]. Scores range from 0 to 60, with higher scores indicating greater coparenting.

We will also assess the implementation of Essential Coaching Postpartum using output data from the Twilio and TextIt platforms (eg, the number of SMS text messages sent, the number of SMS text messages delivered, error messages, unsubscription rates, and link clicks) as well as open-ended feedback from participants collected at 6 weeks post partum.

### Data Analysis

Data will be analyzed on an intention-to-treat basis. Demographic characteristics, including parental age, marital status, educational level, and delivery method (eg, vaginal or cesarean), will be summarized using means and SDs or percentages, as applicable. Postnatal health care contacts for the parent and infant, health concerns, and feeding behavior will also be reported using means and SDs or percentages, as applicable. Any significant differences in baseline characteristics, assessed using chi-square tests or Mann-Whitney *U* tests, will be adjusted for in subsequent analyses. A *P* value of <.05 will be considered statistically significant for all outcomes.

For the primary and secondary outcomes, analysis of covariance will be used to determine whether scores differ between the 2 groups, with adjustments for pretest scores and any significant baseline characteristics. For the exploratory analysis, dyadic actor-partner interdependence multivariate multilevel modeling will be used to examine each dyad member’s relationship to primary and secondary outcomes after accounting for other explanatory variables. For implementation data, quantitative responses will be reported, and thematic analysis [48] will be conducted to describe participants’ experiences with Essential Coaching Postpartum.

## Results

Study recruitment began in June 2025 and is anticipated to be completed within 12 months. As of September 15, we had enrolled 11 birthing parents and 5 nonbirthing parents.

## Discussion

### Anticipated Findings or Significance

We anticipate that providing parents with SMS text messages during the postpartum period to complement standard care will improve parental outcomes. Specifically, we expect that parents (both birthing and nonbirthing) who receive Essential Coaching Postpartum will demonstrate higher parenting self-efficacy and lower depression, anxiety, and stress scores at 6 weeks post partum and 6 months post partum than those who receive standard care. We also anticipate improvements for parents in general distress, sleep, relationship satisfaction, and coparenting outcomes. In addition, for parent dyads in which both members receive Essential Coaching Postpartum, we expect higher parenting self-efficacy, greater relationship satisfaction, and stronger coparenting scores as well as lower depression, anxiety, and stress scores at 6 weeks post partum compared to dyads who receive standard care.

### Comparison to Previous Work

Building on the previous success of Essential Coaching for Every Mother, the Essential Coaching Postpartum program expands to provide both birthing and nonbirthing parents with postpartum support during the immediate 6-week postpartum period. Essential Coaching for Every Mother was evaluated in a feasibility study [26] and an RCT [25], both of which found that it improved parenting self-efficacy and reduced postpartum anxiety, particularly among first-time parents. Birthing parents were also extremely pleased with the program, with most willing to recommend it to other parents. Since its development in 2019 and evaluation in 2021, modifications have been made, including updates based on the 2023 *Loving Care* book series [28,29], as well as the creation of a nonbirthing partner version, Essential Coaching for Every Partner [27].

While other digital health solutions exist for new parents, the Essential Coaching Postpartum SMS text messaging program is unique. It differs from other digital health solutions in three important ways: (1) it is based on Nova Scotia–specific information, is aligned with the *Loving Care* book series [28,29], and was reviewed by Nova Scotian parents and postpartum health care providers; (2) it focuses more on the provision of information within SMS text messages through longer texts (approximately 800 characters), in addition to offering links to local resources; and (3) it provides parental and newborn care information related to the immediate 6-week postpartum period to both parents, with twice-daily text messages in the first 3 weeks and daily messages in the next 3 weeks. Overall, Essential Coaching Postpartum is designed specifically for the immediate postpartum period and tailored to parents in Nova Scotia, while also providing individualized, evidence-based support for both birthing and nonbirthing parents.

Other perinatal digital health solutions in Canada include SmartMom and SmartParent, which are SMS text messaging programs that provide support for parents during pregnancy and through their child’s first year of life. These programs were designed by academic researchers from the University of British Columbia in collaboration with the British Columbia Ministry of Health, British Columbia health authorities, and Perinatal Services BC and in consultation with pregnant individuals and new parents, nurses, physicians, midwives, and experts in maternal and child health [49]. The programs provide 3 messages per week from conception through 1 year post partum, containing evidence-based information and links to support healthy pregnancy, childbirth, transition to parenthood, and infant growth and development [50]. To date, SmartParent has not been evaluated for its impact on parenting self-efficacy or postpartum mental health; however, a protocol was recently published to evaluate SmartMom’s impact on prenatal outcomes [51].

Another existing digital health solution for nonbirthing parents is SMS4Dads, which is an SMS text messaging program developed to support paternal mental health in Australia [52]. SMS4Dads sends fathers SMS text messages from pregnancy through 6 months post partum, focusing on paternal mental and physical health, strategies for building a strong and nurturing relationship with their child, and approaches to developing a strong coparenting relationship with their partner [52]. In a feasibility study involving 520 fathers over 8 months, two-thirds of the fathers accessed the resource links provided in the SMS text messages, and all reported that the messages promoted a positive transition to fatherhood [52]. However, this program is available only in Australia, with future research ongoing to determine its effectiveness [53].

### Future Directions

This study will (1) advance understanding of the effectiveness of a parent-targeted intervention for improving parental self-efficacy, mental health, well-being, and parenting outcomes; (2) examine the utility of providing an evidence-based intervention via SMS text messaging; (3) investigate the uptake of a postpartum mental health prevention intervention for parents; and (4) improve the health of families in Nova Scotia, complementing existing care, by closing gaps in standard care offered during the postpartum period.

As next steps, we will first expand the reach of the Essential Coaching Postpartum program by translating it into additional languages, including French, Arabic, and simplified Chinese. Second, we will conduct an implementation study to determine how we can implement the program across Nova Scotia. Finally, we aim to expand to other provinces in Canada, ensuring that the content remains consistent with local postpartum education for both birthing and nonbirthing parents.

## Appendix

Multimedia Appendix 1 Peer review report by IWK Scientific Review Committee, Research and Innovation Advancement, IWK Health Centre (Nova Scotia, Canada).

Multimedia Appendix 2 CONSORT-eHEALTH checklist (V 1.6.1).

## Abbreviations

CONSORT-EHEALTH: Consolidated Standards of Reporting Trials of Electronic and Mobile Health Applications and Online Telehealth
PROMIS: Patient-Reported Outcomes Measurement Information System
RCT: randomized controlled trial
REDCap: Research Electronic Data Capture

## References

[1] Walker SB, Rossi DM, Sander TM (2019). Women's successful transition to motherhood during the early postnatal period: a qualitative systematic review of postnatal and midwifery home care literature. Midwifery.

[2] Kowlessar O, Fox JR, Wittkowski A (2014). First-time fathers’ experiences of parenting during the first year. J Reprod Infant Psychol.

[3] McKelvey MM (2014). The other mother: a narrative analysis of the postpartum experiences of nonbirth lesbian mothers. ANS Adv Nurs Sci.

[4] Paulson JF, Bazemore SD (2010). Prenatal and postpartum depression in fathers and its association with maternal depression: a meta-analysis. JAMA.

[5] Ross LE (2005). Perinatal mental health in lesbian mothers: a review of potential risk and protective factors. Women Health.

[6] Bandura A (1977). Self-efficacy: toward a unifying theory of behavioral change. Psychol Rev.

[7] Fang Y, Boelens M, Windhorst DA, Raat H, van Grieken A (2021). Factors associated with parenting self-efficacy: a systematic review. J Adv Nurs.

[8] Aston M, Price S, Monaghan J, Sim M, Hunter A, Little V (2018). Navigating and negotiating information and support: experiences of first-time mothers. J Clin Nurs.

[9] Liu X, Wang S, Wang G (2022). Prevalence and risk factors of postpartum depression in women: a systematic review and meta-analysis. J Clin Nurs.

[10] Cameron EE, Sedov ID, Tomfohr-Madsen LM (2016). Prevalence of paternal depression in pregnancy and the postpartum: an updated meta-analysis. J Affect Disord.

[11] Goodman JH, Watson GR, Stubbs B (2016). Anxiety disorders in postpartum women: a systematic review and meta-analysis. J Affect Disord.

[12] Leiferman JA, Farewell CV, Jewell J, Walls J, Harnke B, Paulson JF, Rachael Lacy (2021). Anxiety among fathers during the prenatal and postpartum period: a meta-analysis. J Psychosom Obstet Gynaecol.

[13] Lebow JL, Chambers AL, Christensen A, Johnson SM (2012). Research on the treatment of couple distress. J Marital Fam Ther.

[14] Yang X, Ke S, Gao L (2020). Social support, parental role competence and satisfaction among Chinese mothers and fathers in the early postpartum period: a cross-sectional study. Women Birth.

[15] Healthy babies, healthy families program to support prenatal and parenting needs. Reproductive Care Program of Nova Scotia.

[16] Dol J, Hughes B, Tomblin Murphy G, Aston M, McMillan D, Campbell-Yeo M (2022). Canadian women's experience of postnatal care: a mixed method study. Can J Nurs Res.

[17] Tully KP, Stuebe AM, Verbiest SB (2017). The fourth trimester: a critical transition period with unmet maternal health needs. Am J Obstet Gynecol.

[18] Spelke B, Werner E (2018). The fourth trimester of pregnancy: committing to maternal health and well-being postpartum. R I Med J (2013).

[19] Hodgson S, Painter J, Kilby L, Hirst J (2021). The experiences of first-time fathers in perinatal services: present but invisible. Healthcare (Basel).

[20] mHealth: New horizons for health through mobile technologies. World Health Organization.

[21] Walker LO, Mackert MS, Ahn J, Vaughan MW, Sterling BS, Guy S, Hendrickson S (2017). e-Health and new moms: contextual factors associated with sources of health information. Public Health Nurs.

[22] van den Heuvel JF, Groenhof TK, Veerbeek JH, van Solinge WW, Lely AT, Franx A, Bekker MN (2018). eHealth as the next-generation perinatal care: an overview of the literature. J Med Internet Res.

[23] Da Costa D, Zelkowitz P, Letourneau N, Howlett A, Dennis C, Russell B, Grover S, Lowensteyn I, Chan P, Khalifé S (2017). HealthyDads.ca: what do men want in a website designed to promote emotional wellness and healthy behaviors during the transition to parenthood?. J Med Internet Res.

[24] Dol J, Tomblin Murphy G, Aston M, McMillan D, Campbell-Yeo M (2021). Design, development and usability testing of essential coaching for every mother: a postnatal text message educational intervention. Women Birth.

[25] Dol J, Aston M, Grant A, McMillan D, Tomblin Murphy G, Campbell-Yeo M (2022). Effectiveness of the "Essential Coaching for Every Mother" postpartum text message program on maternal psychosocial outcomes: a randomized controlled trial. Digit Health.

[26] Dol J, Aston M, Grant A, McMillan D, Tomblin Murphy G, Campbell-Yeo M (2022). Implementing essential coaching for every mother during the COVID-19 pandemic: a pre-post intervention study. Birth.

[27] Dol J, Chambers CT, Parker JA (2025). Developing 'essential coaching for every partner', a postpartum text-message digital health solution for non-birthing parents. Midwifery.

[28] Loving care - birth to 6 months. Parent Health Education Resource Working Group.

[29] Loving care: parents and families. Nova Scotia Health.

[30] Breastfeeding basics. Nova Scotia Department of Health and Wellness.

[31] How to feed your baby with infant formula. Nova Scotia Department of Health.

[32] Dol J, Aston M, McMillan D, Tomblin Murphy G, Campbell-Yeo M (2021). Effectiveness of a postpartum text message program (essential coaching for every mother) on maternal psychosocial outcomes: protocol for a randomized controlled trial. JMIR Res Protoc.

[33] Harris PA, Taylor R, Thielke R, Payne J, Gonzalez N, Conde JG (2009). Research electronic data capture (REDCap)--a metadata-driven methodology and workflow process for providing translational research informatics support. J Biomed Inform.

[34] Home page. TextIt.

[35] NS births and deaths with rates and natural increase. Nova Scotia.

[36] Public health parenting supports. Nova Scotia Health.

[37] Khan A (2023). Newborns being directed to mobile clinics amid doctor shortage. CBC News.

[38] Palmeter P (2024). Number of Nova Scotians in need of family doctor reaches highest level yet. CBC News.

[39] Home page. Twilio.

[40] Crncec R, Barnett B, Matthey S (2008). Karitine parenting confidence scale - manual. Sydney South West Area Health Service.

[41] Cox JL, Holden JM, Sagovsky R (1987). Detection of postnatal depression. Development of the 10-item Edinburgh Postnatal Depression Scale. Br J Psychiatry.

[42] Fallon V, Halford JC, Bennett KM, Harrold JA (2016). The Postpartum Specific Anxiety Scale: development and preliminary validation. Arch Womens Ment Health.

[43] Dennis CL, Brown HK, Brennenstuhl S (2018). Development, psychometric assessment, and predictive validity of the postpartum childcare stress checklist. Nurs Res.

[44] Loviond S, Lovibond P (1995). Manual for the Depression Anxiety Stress Scales. 2nd edition.

[45] Yu L, Buysse DJ, Germain A, Moul DE, Stover A, Dodds NE, Johnston KL, Pilkonis PA (2011). Development of short forms from the PROMIS™ sleep disturbance and sleep-related impairment item banks. Behav Sleep Med.

[46] Feinberg ME, Brown LD, Kan ML (2012). A multi-domain self-report measure of coparenting. Parent Sci Pract.

[47] Funk JL, Rogge RD (2007). Testing the ruler with item response theory: increasing precision of measurement for relationship satisfaction with the Couples Satisfaction Index. J Fam Psychol.

[48] Braun V, Clarke V (2006). Using thematic analysis in psychology. Qual Res Psychol.

[49] Care providers. SmartParent.

[50] What is SmartParent?. SmartParent.

[51] Janssen P, Lecke S, Renner R, Zhang W, Vedam S, Norman WV, Bayrampour H, Tough S, Murray J, Muhajarine N, Dennis CL (2024). Teaching by texting to promote positive health behaviours in pregnancy: a protocol for a randomised controlled trial of SmartMom. BMJ Open.

[52] Fletcher R, Kay-Lambkin F, May C, Oldmeadow C, Attia J, Leigh L (2017). Supporting men through their transition to fatherhood with messages delivered to their smartphones: a feasibility study of SMS4dads. BMC Public Health.

[53] Fletcher R, May C, Attia J, Garfield CF, Skinner G (2018). Text-based program addressing the mental health of soon-to-be and new fathers (SMS4dads): protocol for a randomized controlled trial. JMIR Res Protoc.

