# Supplementary material for: Effectiveness of the Essential Coaching Postpartum Digital Health Solution on Parenting Self-Efficacy, Mental Health, Well-Being, and Parenting Outcomes: Protocol for a Randomized Controlled Trial
Source: JMIR Res Protoc. 2025 Oct 17;14:e78209. doi: 10.2196/78209 (PMC12579292; doi:10.2196/78209)
Supplement: Multimedia Appendix 1 [file resprot_v14i1e78209_app1.pdf]

Research & Innovation Advancement
IWK Health

Research & innovation Advancement
5850/5980 University Avenue
PO Box 9700, Halifax
Nova Scotia | B3K 6R8
Canada

December 9, 2024

To: Justine Dol, Christine Chambers and Jennifer Parker

From: Scientific Officer of the IWK Scientific Review Committee

Re: 1031109 Study Title: Essential Coaching for Every Parent: Evaluating a parent-focused postpartum text message program in Nova Scotia

The following is a summary of the discussions that took place at the Scientific Review Committee Meeting in relation to your application for funding. These notes highlight additional considerations, not outlined in reviewer feedback, contributing to your final score.

The committee members concurred that the proposed work fits the IWK mandate with a focus on family centered care that could have an impact on family/child health. The work addresses gaps in post-natal care and inclusivity was seen as an added strength. The team is composed of an excellent PI and a highly experienced, well-rounded research team with the right qualifications and experience.

There was some discussion around the recruitment and retention of dyads which could be challenging during an already stressful and tiring time for new parents. From a purely grantsmanship perspective, the reviewers felt that the specific tools being used to assess the identified variables of interest would have been better placed in the methods section.

Research & Innovation Advancement
IWK Health

Research & innovation Advancement
5850/5980 University Avenue
PO Box 9700, Halifax
Nova Scotia | B3K 6R8
Canada

I wish you the best of luck with this work. If you require any further scientific consulting services, please do not hesitate to contact me.

Jill Hatchette, PhD
Consulting Scientist
Research Services
IWK Health Centre
jill.hatchette@iwk.nshealth.ca
5850/5980 University Avenue | PO Box 9700
Halifax, Nova Scotia | B3K 6R8

Applicant: Justine Dol

Co-Applicant: Christine Chambers and Jennifer Parker

Study Title: 1031109 - Essential Coaching for Every Parent: Evaluating a parent-focused postpartum text message program in Nova Scotia

REVIEWER COMMENTS

PRIMARY REVIEWER

Briefly outline the aim(s) and description of the project (*purpose, hypothesis, approach, etc.*)

- Goal: Help make the first few weeks after a new baby less stressful by providing information and support directly to parents that they know they can trust.
- Conduct a randomized controlled trial to evaluate the effectiveness of the Essential Coaching program on parent dyads in Nova Scotia.
- Transition to parenthood is challenging.
- Confidence/anxiety/depression/isolation concerns
- Phone/internet as source of information.
- Using mHealth (mobile health) to provide information directly to parents after the birth of their first baby via the Essential Coaching program.
- 332 parents with text messages sent for 6 weeks after birth to share information on newborn care and parent outcomes.
- Different messages for birthing parent (Essential Coaching for Every Mother) or non-birthing parent (Essential Coaching for Every Partner)
- Compare those who receive the messages to a group of parents do not receive the messages. Compare parents' confidence, anxiety, depression, and co-parenting between the two groups after six-weeks and six-months.
- Hypothesis: Parents who get the text messages will have higher confidence and co-parenting outcomes and lower anxiety and depression.

Investigator Merit: (Do they have the proper qualifications and research experience? Is the proper team in place to complete this research?)

Lead: Dr. Justine Dol, a postdoctoral fellow at IWK Health. PI of the Essential Coaching for Every Mother RCT and led the development of Essential Coaching for Every Parent. Led similar RCTs, responsible for the project oversight and implementation.

Co-investigators include:(1) Dr. Christine Chambers, Professor, Psychology & Neuroscience, clinical psychologist, and Tier 1 Canada Research Chair in Children's Pain, Dr. Dol's postdoctoral supervisor; (2) Dr. Jennifer Parker, Research Scientist, IWK Health with expertise in research methods and statistics, patient engagement, and knowledge translation; (3) Dr. Melissa Brooks, Assistant Professor, Department of Obstetrics and Gynecology, MD, and lead of Smart Parent NS; (4) Dr. Cindy-Lee Dennis, Professor, Nursing, University of Toronto with expertise in perinatal mental health in birthing and non-birthing parents; (5) Dr. Daniel Seguin, Professor, Psychology, Mount Saint Vincent University with expertise in parenting intervention expertise and 2SLGTBQ+ populations; and (6) Dr. Jennifer Goldberg, Postdoctoral Fellow, McMaster Midwifery Research Centre with expertise in critical 2SLGBTQAI health and midwifery scholar
Strong qualifications and experience. Amazing team in place!

Scientific Merit (consider impact/relevance to the IWK, clarity of goals and objectives, appropriateness of methods and analysis, challenges are identified, novelty of the research.)

Evaluate the effect of the Essential Coaching program on parenting self-efficacy, mental health, and parenting outcomes among first-time parents in Nova Scotia. Clear goal.

Impact/relevance to IWK: support and empower the Non-Birthing Parent through information which will enhance engagement; family-centered care; maternal/child health.

- Data will be analyzed using mixed linear regression models.
- No challenges identified.
- Innovative

Strengths:

- Builds on previous work.
- Essential Coaching for Every Mother RCT was funded by an IWK Mentored Grant and IWK Ross Fund
- Development of Essential Coaching for Every Partner was funded by an IWK Mentored Grant
- Now look to evaluate the effectiveness of the Essential Coaching program on parent dyads in Nova Scotia
- Inclusivity non-birthing parent focus, LGBTQ+
- Support and outcome focus.

Weaknesses:

What does the survey look like? How are they evaluating the families experience? What tool is being used? Feel this was a major gap. Perhaps I missed that part?

Budget Considerations: $25,000

- SALARY of Research Assistant: $8,130 (1/3 of costs). Recruitment and data entry. 0.2FTE hours. Not sure length of time?
- Note: Infrastructure and equipment support available through their role as a postdoctoral fellow with Dr. Christine Chambers. Salary support is available through their CIHR Postdoctoral Fellowship (2023-2026). Statistical support is available through the Maritime SPOR Support Unit in their role as a trainee.
- Feel budget is appropriate and properly justified.

<u>SECONDARY REVIEWER</u>

Investigator Merit: (Do they have the proper qualifications and research experience? Is the proper team in place to complete this research?)

Dr. Dol has an impressive track record, with 33 publications since 2017. The co-investigators include Drs. Chambers, Parker, Brook, Dennis, and Seguin—each bringing specialized expertise in relevant areas such as pediatrics, obstetrics, nursing, and psychology. This collaborative team is highly qualified to conduct the proposed research.

Scientific Merit (consider impact/relevance to the IWK, clarity of goals and objectives, appropriateness of methods and analysis, challenges are identified, novelty of the research.)

The study hypothesizes that the intervention will improve self-efficacy, reduce anxiety and depression, and enhance co-parenting compared to standard care. Mixed linear regression models and multilevel modeling will be employed to analyze the data, adjusting for demographic variables and intervention adherence. The

anticipated outcomes suggest that the Essential Coaching intervention could support diverse parenting experiences and enhance parental well-being, particularly for non-birthing parents, who often receive less support in the postpartum period.

This research has the potential to provide valuable insights into the effectiveness of digital interventions in supporting parental mental health and improving co-parenting outcomes during the postpartum period. The findings could have broader implications for health care in Nova Scotia and beyond. **This study is highly relevant to IWK Health, which specializes in maternal, newborn, and pediatric care.**

Strengths:
The study's significance lies in its potential to address gaps in postpartum care, particularly in the context of Canada's lack of standardized support for new parents. The use of digital text messaging is a practical, cost-effective approach that could improve mental health and parenting outcomes without overwhelming the healthcare system.

Weaknesses:
Challenges may arise in recruiting a diverse participant group and ensuring active engagement from both parents in the dyad. However, the remote nature of the intervention and the research team's prior success with similar studies suggest that these challenges are manageable.

Budget Considerations: The proposed budget is adequate to cover the salary of a research assistant, consumables, and recruitment costs for the study.
